# Kinetic Study of Acid Hydrolysis of Rice Straw

**DOI:** 10.5402/2013/170615

**Published:** 2013-12-22

**Authors:** Nibedita Sarkar, Kaustav Aikat

**Affiliations:** Department of Biotechnology, National Institute of Technology, Mahatma Gandhi Avenue, Durgapur 713209, India

## Abstract

Rice straw is a renewable, cheap, and abundant waste in tropical countries. The pentose content of rice straw can be used as a substrate for many types of value-added products such as xylitol and biofuel. Dilute acid hydrolysis mainly releases pentose from rice straw. The objective of the study was to determine the effect of H_2_SO_4_ concentration and reaction time on the xylose production. The variation of the main product xylose with the reaction time was described by a kinetic model and kinetic parameters were calculated to describe the variation of the xylose production with time. The optimum yield (19.35 g/L) was obtained at 0.24 mol/L H_2_SO_4_ and 30 minutes.

## 1. Introduction

Rice straw is one of the most abundant agricultural wastes. For instance, approximately 731 million tons per year rice straw is produced globally (Africa: 20.9 million tons, Asia: 667.6 million tons, Europe: 3.9 million tons, America: 37.2 million tons 61, and Oceania: 1.7 million tons) [1]. Nearly 600 million tons of agricultural residues are produced by India annually; out of which, approximately 300 million tons of it remain unused. 

The options for the disposal of rice straw are limited by the great bulk of material, slow degradation in the soil, harboring of rice stem diseases, and high mineral content. Though rice straw is used as animal feed and soil fertilizer, the utilization ratio remains low compared to other straws. Rice straw has low digestibility value as animal feed. Although rice straws contain materials for social benefit, their apparent value is less than the cost of collection, transportation, and processing for beneficial use. Open-field burning is a major practice for rice straw disposal. It causes air pollution, a threat to human health [2]. Rice straw contains 19–27% of hemicellulose, a heteropolymer composed mainly of xylose followed by arabinose [3, 4]. The hemicellulosic and cellulosic contents of rice straw can be hydrolyzed chemically or enzymatically. Chemical hydrolysis includes dilute sulfuric acid hydrolysis that can be used for either the pretreatment before enzymatic hydrolysis or the conversion of hemicellulose to pentose [5] remaining cellulose and lignin fractions being almost unaltered. Lignocellulosic structure as well as hydrolysis reactions of sugar polymers in a dilute acid medium is very complicated. The substrate is in the solid phase and the catalyst in the liquid phase. Various factors (particle size, liquid to solid ratio, type and concentration of acid used, temperature, and reaction time) influence monomer yield [6].

In the present work, the effect of acid concentration and reaction time on xylose production was studied and a kinetic model was developed to describe the variation of xylose production with time.

## 2. Materials and Methods

### 2.1. Rice Straw

Rice straw was used as raw material in the experiments. It was procured from Bankura, India. It was air-dried, ground, size fractioned to 0.5 mm, and stored at room temperature for subsequent experiments. Main components of rice straw such as cellulose, hemicellulose, and lignin were determined [7]. The composition of rice straw is shown in Table 1.

#### 2.1.1. Cellulose Estimation

Acetic/nitric reagent (150 mL of 80% acetic acid and 15 mL of concentrated nitric acid) was added to 0.5 g sample and placed in a water bath at 100°C for 30 minutes. The mixture was cooled and centrifuged for 20 minutes. The supernatant was discarded and the residue was washed with distilled water and mixed with 10 mL of 67% H_2_SO_4_. Then it was allowed to stand for 1 hour at room temperature. The solution (1 mL) was diluted to 100 mL and to 1 mL of this diluted solution and 10 mL of freshly prepared anthrone reagent (0.2% anthrone in concentrated H_2_SO_4_) was added. The mixture was heated in a boiling water bath for 10 minutes and cooled. The color was measured at 630 nm. Cellulose powder (Hi Media, India) was used as a standard. 

#### 2.1.2. Hemicellulose Estimation

In a refluxing flask, 10 mL cold neutral detergent solution was added to 1 g powdered sample. The neutral detergent solution was prepared as follows. Disodium ethylenediamine tetraacetate (18.61 g) and sodium borate decahydrate (6.81 g) were dissolved in about 200 mL of distilled water by heating and to this, 100 mL solution containing sodium lauryl sulphate (30 g) and ethoxy ethanol (10 mL) was added. A solution (100 mL) of 4.5% Na_2_HPO_4 _ was then added to the mixture. The final volume was made up to 1 L with distilled water and the pH adjusted to 7. 

To the mixture of rice straw sample and cold neutral detergent solution, decahydronaphthalene (2 mL) and sodium sulphite (0.5 g) were added. Then the mixture was heated to boiling and refluxed for 1 h. The contents inside the refluxing flask were filtered through sintered glass crucible (G-2) followed by hot water washing. Finally two washings with acetone were given and the residue was transferred to a crucible. The sample was dried at 100°C for 8 h, cooled in a desiccator, and weighed. The residue was designated as neutral detergent fiber (NDF). The amount of acid detergent fiber (ADF) was subtracted from the amount of neutral detergent fiber (NDF) for the calculation of hemicellulose content. The acid detergent fiber (ADF) was prepared by the following method.

100 mL acid detergent solution (2% cetyl trimethyl ammonium bromide in 1 N sulphuric acid) was added to the powdered sample which was placed in a round bottom flask. The sample was heated to boil in 5–10 minutes and refluxed for 1 h after the onset of boiling. The boiling was adjusted to slow, even level. The contents were filtered through a preweighed sintered glass crucible (G-2) by suction and washed with hot water twice. The contents were washed with acetone until the filtrate was colorless. Then it was dried at 100°C for overnight, cooled in a desiccator, and weighed. The ADF content was expressed as a percentage, that is, *W*/*S* × 100 where *W* was the weight of the fiber and *S* was the weight of the sample.

#### 2.1.3. Lignin Estimation

25 mL of 72% H_2_SO_4_ and 1 g asbestos were added to acid detergent fiber (ADF). The mixture was kept for 3 h at room temperature and intermittently stirred. After that the mixture was diluted and filtered with preweighed Whatman no. 1 filter paper. Then it was dried at 100°C, cooled in a desiccator and weighed. Then the filter paper was transferred to a preweighed silica crucible and kept in a muffle furnace at 550°C for 3 h. The crucible was cooled in a desiccator, and weighed for ash content calculation. Asbestos was used as blank. The acid detergent lignin (Percentage of ADL) was determined using

(1) Percentage of  ADL =  Weight  72%  H2SO4  washed  fibre  (test−asbestos  blank)  ×100(Weight  of  the  sample)−1  −Ash(test−asbestos  blank)×100Weight  of  the  sample.

### 2.2. Acid Hydrolysis

The basic method of acid hydrolysis of rice straw by diluted H_2_SO_4_ at 121°C and 15 psi was done as per Yoswathana et al., 2010 [8]. Rice straw (1 g) was treated with 10 mL of H_2_SO_4_ solution of various concentrations for different reaction times. At given time intervals, samples were withdrawn, cooled, and centrifuged at 12000 rpm for 15 minutes to separate out the water insoluble fraction. The supernatant was used for xylose assay. All the hydrolysis experiments were carried out in triplicate.

### 2.3. Analytical Methods

#### 2.3.1. Xylose Estimation

Xylose content was determined using the phloroglucinol assay [9] with minor modifications. Briefly, the color reagent consisting of 0.5 g of phloroglucinol, 100 mL of glacial acetic acid, and 10 mL of conc. HCl was freshly prepared and used within 4 days. Stock of standard xylose solution (10 g/L) was prepared by dissolving D-xylose (Himedia, India) powder in saturated benzoic acid and used for preparation of the calibration curve. Fifty microliters of sample was mixed with 5 mL color reagent and subsequently heated at 100°C for 6 min. The reaction mixture was rapidly cooled down to room temperature in a water bath and the absorbance at 554 nm was recorded.

## 3. Results and Discussion

For acid hydrolysis, H_2_SO_4_ concentration was varied from 0.093 mol/L to 0.28 mol/L and reaction time was varied from 15 to 60 minutes. Xylose concentration obtained from the hydrolysate at various H_2_SO_4_ concentrations and reaction times is given in Table 2. It was observed that maximum xylose was released at 0.24 mol/L H_2_SO_4_ concentration and 30 minutes. Xylose concentration decreased with reaction time. This may be due to sugar decomposition and production of inhibitor. The level and composition of the sugar released depend on the type of the acid in the reaction mixture [10] and acid treatment with H_2_SO_4_ mainly releases xylose [11].

The variation of the main product xylose with reaction time may be described by a kinetic model. Saeman, 1945 [12], proposed a simplified model for hydrolysis of lignocellulosic materials using pseudo-homogeneous irreversible first-order reactions [13, 14]. The model was used for hemicellulosic fraction [13]:

(2) $$\begin{array}{c} \text{polymers}\overset{k_{1\;\;\;\;\;}}{\to }\text{monomers}\overset{k_{2\;\;\;\;\;}}{\to }\text{decomposition}, \end{array}$$

where *k*_1_ and *k*_2_ are the rate constants (min^−1^) for generation and decomposition reaction, respectively.

Predicted model for concentration of monomers is given below by solving the differential equation [12]:

(3) $$\begin{array}{c} M=M_{0}e^{-k_{2}t}+P_{0}\frac{k_{1}}{k_{2}-k_{1}}\left(e^{-k_{1}t}-e^{-k_{2}t}\right), \end{array}$$

where *M* is concentration of monomers (g/L), *P* is concentration of polymers (g/L), *M*_0_ is initial monomer concentration ( = 0), and *t* is reaction time.

Equation (3) is used for kinetic modeling of xylose concentration.

*P*_0_ of (3) is calculated as follows:

(4) $$\begin{array}{c} P_{0}=\frac{150}{132}\frac{C_{Xn_{0}}}{\text{WSR}}\times 10=23.4\;\text{g}\;\text{eq}.\text{xylose}/\text{L}, \end{array}$$

where *C*_*Xn*_0__ initial xylan concentration was assumed as 20.6 g xylan per 100 g rice straw on dry basis [15]. WSR is the water/solid ratio and 150/132 is the ratio of the stochiometric factors.

Aguilar et al. (2002) [13] modified (3) for better fitting of the experimental data to the kinetic model. The modified equation is given by

(5) $$\begin{array}{c} M=M_{0}e^{-k_{2}t}+\alpha P_{0}\frac{k_{1}}{k_{2}-k_{1}}\left(e^{-k_{1}t}-e^{-k_{2}t}\right). \end{array}$$

**Figure 1:**
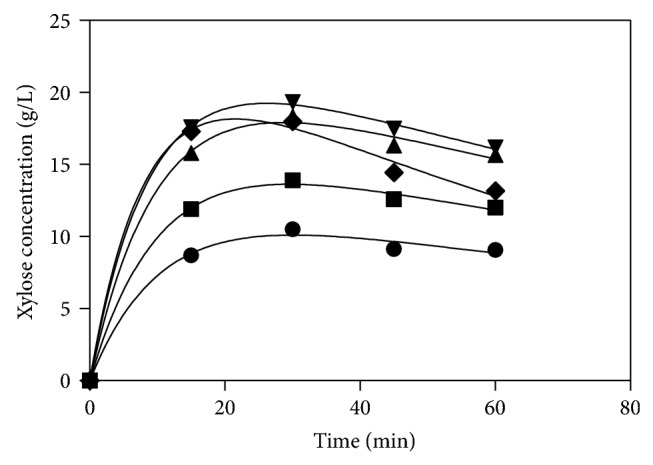
Experimental and predicted dependence of xylose concentration on time of various H_2_SO_4_ concentrations. (—) Saeman's model, (●) 0.093 mol/L, (▪) 0.14 mol/L, (▲) 0.18 mol/L, (*▼*) 0.24 mol/L, and (*◆*) 0.28 mol/L.

*α* is the ratio between fractions (g of susceptible xylan/g of total xylan). The usual range of *α* is 0.5–1 g/g. Experimental data of xylose concentration in acid hydrolysate was fitted applying (5). The plots of predicted and experimental data of xylose concentration of each set are shown in Figure 1. The kinetic parameters of each set are given in Table 3. Values of correlation coefficient *R* ^2^ for each set were 0.99 which indicate the fitness of the model. 

Chi-square analysis of the experimental data at each H_2_SO_4_ concentration was carried out. The chi-square statistic test (*χ* ^2^) is the sum of the squares of the differences between the experimental data and predicted data obtained by calculating from models, with each squared difference divided by the corresponding predicted data obtained by calculating from models. The (*χ* ^2^) will be a small number if the experimental data and predicted data from the model are similar and vice versa. Therefore, the model that gives the smallest chi-square value is considered the best fit [16]:

(6) $$\begin{array}{c} \chi^{2}\;=\;\sum \frac{{\left(q_{e,\exp}\;-\;q_{e,\text{cal}}\right)}^{2}}{q_{e,\text{cal}}}. \end{array}$$

The range of the *χ* ^2^ value signifies the good fit of the model at each acid concentration (Table 4). 

From Table 3, it is observed that *α* values varied from 0.528 to 0.999 depending on H_2_SO_4_ concentration. Values of *α* generally vary with operational conditions. Different *α* values ranging from 0.5 to 1 g/g have been reported [13].

Values of *k*_1_ and *k*_2_ values also increased with H_2_SO_4_ concentration and *k*_1_ values were higher compared to *k*_2_ values for each set. This indicates that generation rate is higher than degradation rate. 

For zero initial monomer, rate of monomer formation *dM*/*dt* can be obtained by differentiating (5) and putting *M*_0_ = 0:

(7) $$\begin{array}{c} \frac{dM}{dt}=\alpha P_{0}\frac{k_{1}}{k_{2}-k_{1}}\left(-k_{1}e^{-k_{1}t}+k_{2}e^{-k_{2}t}\right). \end{array}$$

Now for heterogeneous solid-liquid noncatalytic reactions,

(8) $$\begin{array}{c} \frac{dM}{dt}=\text{kFC}, \end{array}$$

where *k* is mass transfer constant, *F* is interfacial area, and *C* is acid concentration. Comparing (7) and (8), the following equation can be obtained:

(9) $$\begin{array}{c} \text{kFC}=\alpha P_{0}\frac{k_{1}}{k_{2}-k_{1}}\left(-k_{1}e^{-k_{1}t}+k_{2}e^{-k_{2}t}\right). \end{array}$$

**Figure 2:**
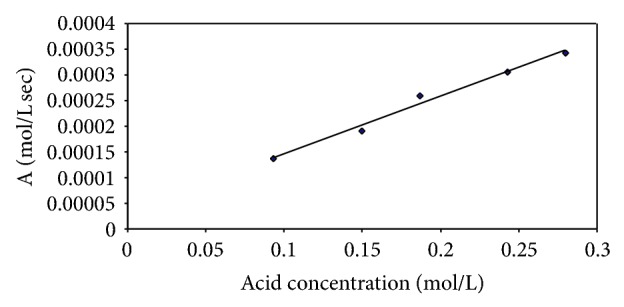
A plot of  *αP*_0_(*k*_1_/(*k*_2_ − *k*_1_))  (−*k*_1_ *e* ^−*k*_1_*t*^ + *k*_2_ *e* ^−*k*_2_*t*^) (denoted by “A”) versus C (acid concentration).

A plot of  *αP*_0_(*k*_1_/(*k*_2_ − *k*_1_))  (−*k*_1_ *e* ^−*k*_1_*t*^ + *k*_2_ *e* ^−*k*_2_*t*^) (denoted by “A”) versus *C* (acid concentration) should give a straight line. The results obtained were in agreement with this as shown in Figure 2. Linear regression of (9) gave an *R* ^2^ of 0.98 assuming *k* and *F* to be nearly constant.

A short discussion of the practical utility of the present work may be relevant here. The optimized xylose production obtained in the present study (19.35 g/L) was higher than in previous reports [14, 17]. For this level of production at the laboratory scale, the raw material cost was computed to be approximately $ 1.5 per kg xylose. This cost is quite low enough to indicate the economic feasibility of the process on the large scale. 

Now acid hydrolysis breaks down mainly the hemicellulose component of rice straw to release the pentose, mainly xylose, in the hydrolysate. Acid hydrolysis can also bring about delignification of the straw to make the cellulosic component amenable to saccharification to release hexoses such as glucose [15]. This saccharification may be carried out enzymatically or by second step acid hydrolysis [15]. Now the first step of acid hydrolysis cannot be prolonged to release both pentoses and hexoses together as excessive treatment time decomposes the pentoses which are released first. 

Regarding the utilization of rice straw, an agricultural waste, for production of value-added products, both pentose and hexose sugars are useful substrates for bioethanol production. Also, xylose can be used for xylitol production. Hence, xylose production is an important component in the complete utilization of rice straw.

## Figures and Tables

**Table 1:** Main components of rice straw.

Components	Percentage of dry weight
Cellulose	40.09 ± 0.02
Hemicellulose	26.8 ± 0.02
Lignin	18.9 ± 0.02

**Table 2:** Xylose (g/L) obtained from the rice straw hydrolysate at various H_2_SO_4_ concentrations and reaction times.

H_2_SO_4_ concentration (mol/L)	Time (min)			
	15	30	45	60
0.093	8.7	10.5	9.13	9.08
0.14	11.9	13.9	12.6	12
0.18	15.8	18.33	16.33	15.65
0.24	17.6	19.35	17.5	16.2
0.28	17.31	17.99	14.45	13.18

**Table 3:** Kinetic parameters of xylose released from rice straw through H_2_SO_4_ hydrolysis.

H_2_SO_4_ concentration (mol/L)	*α* (g/g)	*k*_1_ (min^−1^) × 10^−2^	*k*_2_ (min^−1^) × 10^−3^	*R* ^2^
0.093	0.528	9.4	6	0.993
0.14	0.714	9.6	6.8	0.998
0.18	0.936	9.9	7	0.9973
0.24	0.995	11.1	7.2	0.999
0.28	0.999	12	11.1	0.9958

**Table 4:** Chi-square analysis (*χ* ^2^) at each acid concentration.

H_2_SO_4_ concentration (mol/L)	*χ* ^2^
0.093	0.067
0.14	0.018
0.18	0.035
0.24	0.0084
0.28	0.087
